# A 12-week after-school physical activity programme improves endothelial cell function in overweight and obese children: a randomised controlled study

**DOI:** 10.1186/1471-2431-12-111

**Published:** 2012-07-31

**Authors:** Jong-Hwan Park, Masashi Miyashita, Yoo-Chan Kwon, Hyun-Tae Park, Eun-Hee Kim, Jin-Kee Park, Ki-Beam Park, Suk-Ran Yoon, Jin-Woong Chung, Yoshio Nakamura, Sang-Kab Park

**Affiliations:** 1 Graduate School of Sport Sciences, Waseda University, 2-579-15 Mikajima, Tokorozawa, Saitama 359-1192, Japan; 2 Faculty of Sport Sciences, Waseda University, 2-579-15 Mikajima, Tokorozawa, Saitama, 359-1192, Japan; 3 College of Sport Sciences, Dong-A University, 840 Hadan 2-dong, Saha-gu, Busan 604-714, South Korea; 4 Department of Functioning Activation, National Center for Geriatrics and Gerontology, 35 Gengo, Morioka-machi, Obu-city, Aichi 474-8511, Japan; 5 Cell Therapy Research Center, Korea Research Institute of Bioscience and Biotechnology, 125 Gwahak-ro, Yuseong-gu, Daejeon 306-809, South Korea; 6 College of Natural Sciences, Dong-A University, 840 Hadan 2-dong, Saha-gu, Busan 604-714, South Korea

**Keywords:** After-school exercise programme, Carotid intima-media thickness, Endothelial cell function, Endothelial progenitor cells, Overweight/obese children

## Abstract

**Background:**

Endothelial dysfunction is associated with childhood obesity and is closely linked to the amount and function of endothelial progenitor cells. However, it remains unclear whether endothelial progenitor cells increase with after-school exercise in overweight and obese children. The purpose of this study was to investigate the effects of an after-school exercise programme on endothelial cell function in overweight and obese children.

**Methods:**

A total of 29 overweight/obese children (12.2 ± 0.1 years) were randomly divided into control (i.e. no after-school exercise, n = 14) and after-school exercise (n = 15) groups. The 12-week after-school exercise intervention consisted of 3 days of combined aerobic and resistance exercise per week. Each 80-minute exercise programme included 10 minutes of warm-up and 10 minutes of cool-down after school. CD34^+^ (a cell surface marker on hematopoietic stem cells), CD133^+^ (a cell surface marker on hematopoietic progenitor cells) and CD34^+^/CD133^+^ (considered as endothelial progenitor cells) were measured at baseline and after 12 weeks using flow cytometry.

**Results:**

Increased percentages of CD34^+^, CD133^+^ and CD34^+^/CD133^+^ cells were observed in the after-school exercise group (*p* = 0.018; *p* = 0.001; *p* = 0.002, respectively) compared with the control group. Carotid intima-media thickness decreased after 12 weeks in the after-school exercise group (*p* = 0.020) compared with the control group.

**Conclusions:**

This study provides preliminary evidence that a combined after-school exercise programme may represent an effective intervention strategy for improving vascular repair and endothelial cell function, leading to improved cardiovascular health in overweight and obese children.

**Trial registration:**

Current Controlled Trials ISRCTN19037201

## Background

Le and colleagues have reported that the arteries of obese children could be just as plaque as those of middle-aged people [1]. This build-up could put children at risk for strokes or cardiovascular disease as early as age 30. Furthermore, the study of Le and colleagues found that the carotid artery intima-media thicknesses of obese children were similar to those of a healthy 45-year-old, and these children had high levels of LDL and triacylglycerol and low levels of HDL [1]. Therefore, lifestyle changes and preventative measures are necessary to reduce and prevent childhood obesity.

Emerging evidence supports a role for bone marrow-derived circulating endothelial progenitor cells in maintaining endothelial function and organ perfusion by mechanisms ranging from endothelial repair to postnatal angiogenesis and vasculogenesis [2]. It has been previously demonstrated that the reduction and dysfunction of circulating endothelial progenitor cells are strongly associated with the development of atherosclerosis and cardiovascular disease [3,4]. Moreover, increased adiposity is related with a reduced endothelial progenitor cells to release proangiogenic cytokines and resist apoptosis [5]. Also, a previous study has showed that endothelial progenitor cells were inversely correlated with carotid intima-media thicknesse in a middle-aged general population [6]. Therefore, endothelial progenitor cells are an independent predictor of early subclinical atherosclerosis [6].

Obesity in childhood and adolescent is independently related with arterial endothelial dysfunction [7] and is closely linked to the amount and function of endothelial progenitor cells [8]. Meanwhile, regular physical exercise has been shown to mobilise endothelial progenitor cells from bone marrow [9], and aerobic exercise training improves endothelial progenitor cells [10], which have the potential to participate in neovascularisation and repair of damaged endothelium. Nonetheless, it is important to recognise the role that strength and resistance training can have in promoting health and regulating weight in overweight and obese children. Resistance training can improve several other physiologic parameters related to metabolic health, such as total and regional body composition, blood pressure and HDL [11]. Therefore, a resistance training programme was included in a multidisciplinary weight management programme for overweight and obese children [12]. However, it remains unknown whether or not these two different modes of exercise may positively contribute to increase in endothelial progenitor cells.

Therefore, the purpose of this study was to investigate the effects of an after-school aerobic and resistance exercise programme on endothelial progenitor cells in overweight and obese children. We hypothesised that a regular exercise programme for overweight children would elevate and improve the function of circulating endothelial progenitor cells.

## Methods

### Participants

The participants were recruited through a randomised controlled trial in one local elementary school. To be included in this study, the children had to be between 12 and 13 years old, overweight or obese, apparently healthy and not on any medication, and attending regular school. Overweight and obese were defined as having a body mass index ≥the 85^th^ percentile for age and gender, according to the WHO mass index cut off point [13]. A total of 29 boys and girls were randomly divided into the control (n = 14) or exercise (n = 15) groups. However, the number of boys and girls are attempted to match in each group. Exercise group were assigned to a 12-week supervised after-school combined exercise. Participants in the control group were advised to maintain their usual activities of daily living during the study. All participants of the exercise group attended each after-school exercise progamme session and none of the participants dropped out during duration of the intervention. The physical characteristics of the participants are shown in Table 1. This study was approved by the ethics committee of Dong-A University, and written informed consent was obtained from the parents. We based the sample size that we used in our study (control; n = 14, exercise; n = 15) on previously published work [14-16]. In addition, our power calculation assumed at least a 70% increase in CD34^+^, CD133^+^ and CD34^+^/CD133^+^, with a standard deviation of 50%. For a required power of 0.9 (90%), using a two-sample t-test for comparisons, a sample size of at least 8 in each group was required.

**Table 1:** Body composition and maximal oxygen uptake measured at baseline and after 12 weeks

		**Exercise Group (n = 15)**	**Control Group (n = 14)**	** *p* -value (Interaction)**
Age (years)		12.1 ± 0.1	12.2 ± 0.1	N/A
Sex, boys/girls		7/8	7/7	N/A
Overweight/obese	Baseline	7/8	6/8	N/A
	12 weeks	10/5	5/9	
Height (m)	Baseline	1.46 ± 0.02	1.47 ± 0.02	0.905
	12 weeks	1.48 ± 0.02	1.49 ± 0.02	
Body mass (kg)	Baseline	52.0 ± 1.8	52.3 ± 1.3	0.001
	12 weeks	51.0 ± 1.6	54.8 ± 1.3	
BMI (kg/m^2^)	Baseline	24.4 ± 0.4	24.3 ± 0.3	0.001
	12 weeks	23.2 ± 0.4	24.6 ± 0.4	
Waist	Baseline	82.5 ± 1.7	76.3 ± 1.9	0.001
Circumference (cm)	12 weeks	79.9 ± 1.9	78.9 ± 2.0	
SBP (mm Hg)	Baseline	108 ± 3	110 ± 2	0.470
	12 weeks	111 ± 3	111 ± 2	
DBP (mm Hg)	Baseline	62 ± 1	66 ± 2	0.663
	12 weeks	63 ± 2	66 ± 2	
Maximal oxygen	Baseline	34.1 ± 1.5	35.9 ± 1.0	0.001
Uptake (ml/kg/min)	12 weeks	37.8 ± 1.6	33.2 ± 1.0	

Values are mean ± SEM. BMI, body mass index; SBP, systolic blood pressure; DBP, diastolic blood pressure; N/A, not applicable.

### Laboratory measurements

Physical and anthropometric variables were measured at baseline and after 12 weeks in both groups. Body mass and height were measured to the nearest 0.1 kg and 0.1 cm, respectively, using a Venus 5.5 body composition analyser (Jawon Medical, Gyeongsan, Korea). Body mass index was calculated as weight in kilograms divided by the square of the height in meters. Waist circumference was measured to the nearest 0.1 cm at the level of the umbilicus using a flexible plastic tape with the participants in the standing position. Arterial blood pressure was measured with a mercury sphygmomanometer after participants had been seated at rest for 10 minutes. All participants were advised to avoid physical activity for 48 hours prior to each measurement.

### Exercise capacity tests

In both group, exercise capacity was measured at baseline and after 12 weeks using an Intertrack 6025 motorised treadmill (Teaha, Anyang, Korea) and a Quark B_2_ gas analysis system (Cosmed, Rome, Italy). The testing consisted of a modified Balke protocol with a speed of 3 mph and an initial grade of 6%, which increased by 2% every 2 minutes until participants became exhausted [17].

### After-school exercise programme

The 12-week exercise programme intervention consisted of 3 days of combined aerobic and resistance exercise per week (i.e., Monday, Wednesday and Friday). Each 80-minute exercise programme included 10 minutes of warm-up activities and 10 minutes of cool-down activities after school. All training sessions were supervised by 2 experienced trainers. Aerobic exercise consisted of 30 minutes of treadmill walking and/or running at 50-70% of the heart rate reserve (HRR). Participants performed the exercise programme for 30 minutes at 50-60% of the HRR during weeks 1 through 6. After week 6, the emphasis was placed on reaching and maintaining an exercise intensity of approximately 60-70% of the HRR for 30 minutes. The target heart rate was continuously monitored using a Polar heart rate monitor (Polar heart monitor; RS400, Finland). Resistance exercise consisted of 2 rotations of a circuit of 7 dynamic exercises with less than 30 seconds of rest between exercises. Briefly, participants were introduced to the exercise equipment and given instruction by trainers on proper lifting form and technique for the dynamic exercises: bench presses, biceps curls, triceps extensions, leg presses, leg extensions, leg curls and calf raises. The 1-repetition maximum was recorded as the maximum resistance that could be lifted throughout the full range of motion (determined in the unweighted position) using good form once. Each participant’s 1-repetition maximum strength was determined for each exercise. After determination of the 1-repetition maximum, participants trained on the same equipment at 60% of their 1-repetition maximum resistance. They performed 2 rotations of a circuit consisting of 8-12 repetitions of 7 dynamic exercises.

### Measurement of carotid intima-media thickness

The carotid intima-media thickness was measured using a B-mode ultrasound and a 10-MHz probe (LOGIQ 3, GE Healthcare, WI, USA). The participants were examined supine with the neck extended and the probe in the anterolateral position. All carotid intima-media thickness measurements were taken in the longitudinal plane at the artery proximal to the adventitia-media junction. After freezing the image, measurements were made using electronic callipers. The maximal thickness of the intima-media width was measured three times, and the mean value was used for statistical purposes [18]. Carotid intima-media thickness analyses were performed by a board-certified sonographer. The test-retest coefficient of variation of carotid intima-media thickness measurements was 0.6%. The intraclass correlation coefficient for repeated measures of the carotid intima-media thickness is 0.6% and for echocardiographic measurements ranges from 0.5 to 0.8% in our laboratory.

### Measurement of endothelial progenitor cells

**Figure 1:**
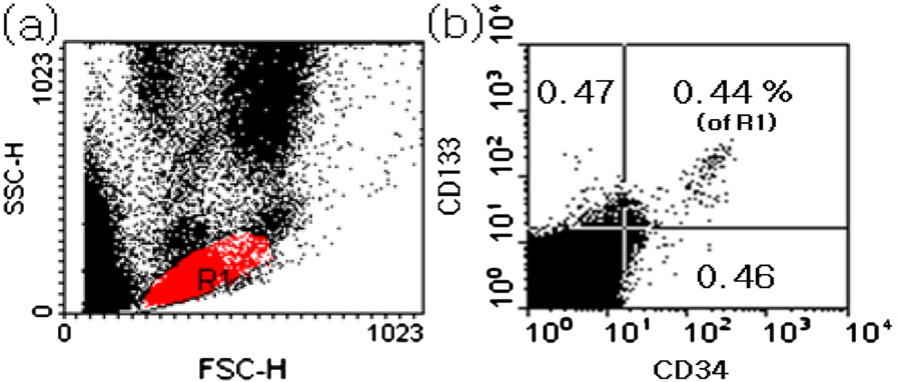
**Gating strategies to detect endothelial progenitor cells by whole blood flow cytometry; one representative blot of endothelial progenitor cells is shown.** (**a**) Forward/sideward scatter with lymphocyte gate (R1) indicated. Acquisition was stopped after 100,000 events acquired in R1. (**b**) Double-positive cells for CD34, CD133 and CD34/CD133 were determined and described as a percentage of the lymphocyte population (R 1).

To measure circulating progenitor cells, lymphocytes were isolated from 5 mL of heparinised whole blood samples and were stained according to a protocol modified from the European Working Group on Clinical Cell Analysis. Vital CD133^+^/CD34^+^ progenitor cells were determined by staining with fluorescein isothiocyanate (FITC)-conjugated anti-CD34 and PE-conjugated anti-CD133 (BD Biosciences, CA, USA). Additional measurements were performed to determine absolute values using True Count beads (BD Biosciences, CA, USA). Fluorescence isotype-matched antibodies were used as controls. Flow cytometry was performed using a FACSCalibur (BD Biosciences, CA, USA). The fluorescence intensities of at least 100,000 cells were recorded and analysed using CellQuest software (BD Biosciences, CA, USA). The combination of CD34^+^ (haematopoietic stem cell maker)/CD133^+^ (haematopoietic progenitor cell maker) is used set for quantification of endothelial progenitor cells in the present study [19]. The percentage of endothelial progenitor cells within the lymphocyte population was defined as events double positive for CD34, CD133 and CD34/CD133 cells. One representative blot of endothelial progenitor cells is presented in Figure 1.

### Other laboratory assays

Fasting venous blood samples were collected from all participants at baseline and at 12 weeks. Participants were advised to avoid physical activity for 48 hours and to fast for at least 10 hours prior to each sample collection (i.e. baseline and 12 weeks). Thus, all samples were collected 48 hours after the last exercise session. All samples were taken at 0830 AM from an antecubital vein. For triacylglycerol, total cholesterol, HDL cholesterol and LDL cholesterol, the samples were collected into tubes containing clotting activators for isolation of serum. Thereafter, samples were allowed to clot for 45 minutes at room temperature and then centrifuged at 3000 rpm for 10 minutes at 4°C. After separation, serum was dispensed into plain micro tubes, and stored at –80°C for later analysis. For plasma insulin, adiponectin, high-sensitivity C-reactive protein, soluble E-selectin, vascular endothelial growth factor and nitric oxide venous blood samples were collected into tubes containing sodium fluoride-EDTA. Thereafter, samples were immediately centrifuged and treated as above. For plasma glucose measurements, venous blood samples were collected into tubes containing sodium fluoride-EDTA. Thereafter, samples were immediately centrifuged and treated as above.

Plasma concentration of nitric oxide was measured using colorimetric assay kits from Neogen (Neogen Inc., MA, USA). Concentrations of plasma vascular endothelial growth factor (IBL Co. Ltd., Gunma, Japan), soluble E-selectin (R&D Systems Inc., MN, USA), high-sensitivity C-reactive protein (R&D Systems Inc., MN, USA), adiponectin (Adipogen Inc., CA, USA) and insulin (Mercodia AB., Uppsala, Sweden) were measured by enzyme-linked immunosorbent assay using commercially available kits. Concentrations of serum total cholesterol, triacylglycerol, HDL cholesterol, LDL cholesterol and plasma glucose were determined by standard laboratory methods.

### Statistical analysis

Data were analysed using Predictive Analytics Software (PASW) version 18.0 for Windows (SPSS Japan Inc., Tokyo, Japan). The unpaired student’s t-test was used to assess group differences in baseline variables. The Shapiro-Wilk test was used to check for normality of distribution. A two-factor analysis of variance (ANOVA) was used to determine interaction (group × time) effects for all outcome variables. Statistical significance was accepted at the 5% level. Results are presented as the means ± SEM.

## Results

The baseline physical characteristics are presented in Table 1. At baseline, the physical characteristics were not significantly different between the two groups.

All participants of the exercise group attended each after-school exercise session (i.e. 36 sessions) and no participants dropped out during the duration of the intervention. Body composition and cardiorespiratory fitness, measured at baseline and after 12 weeks, are presented in Table 2. Two-factor ANOVA revealed group × time interactions for body mass, body mass index, waist circumference and maximal oxygen uptake (*p* = 0.001 for all). Within-group analyses showed that waist circumference measured at 12 weeks was significantly lower than baseline values in the exercise group (P = 0.014), and maximal oxygen uptake measured at 12 weeks was higher than baseline values in the exercise group (P < 0.001). However, in the control group, waist circumference measured at 12 weeks was significantly higher than baseline values (P = 0.006), and maximal oxygen uptake measured at 12 weeks was lower than baseline values (P = 0.005). Analysis of variance indicated that responses differed after 12 weeks between the control and exercise groups. There were no significant differences in height, SBP or DBP (*p* = 0.905; *p* = 0.470; *p* = 0.663, respectively).

**Table 2:** Changes in blood parameters at baseline and 12 weeks

		**Exercise Group (n = 15)**	**Control Group (n = 14)**	** *p* -value (Interaction)**
Triacylglycerol (mmol/L)	Baseline	1.1 ± 0.2	1.0 ± 0.1	0.362
	12 weeks	1.2 ± 0.1	1.3 ± 0.1	
TC (mmol/L)	Baseline	3.8 ± 0.2	3.8 ± 0.1	0.108
	12 weeks	4.0 ± 0.2	4.3 ± 0.2	
HDL-C (mmol/L)	Baseline	1.1 ± 0.1	1.2 ± 0.1	0.379
	12 weeks	1.4 ± 0.1	1.4 ± 0.1	
LDL-C (mmol/L)	Baseline	2.3 ± 0.2	2.3 ± 0.1	0.282
	12 weeks	2.4 ± 0.1	2.5 ± 0.1	
Glucose (mmol/L)	Baseline	4.2 ± 0.2	4.5 ± 0.1	0.805
	12 weeks	4.2 ± 0.1	4.6 ± 0.1	
Insulin (pmol/L)	Baseline	10.3 ± 1.0	12.8 ± 1.8	0.957
	12 weeks	10.9 ± 1.1	13.3 ± 0.9	
Adiponectin (ng/mL)	Baseline	14380 ± 1290	12558 ± 385	0.293
	12 weeks	15950 ± 1731	12704 ± 457	
hs-CRP (mg/dL)	Baseline	0.16 ± 0.04	0.19 ± 0.07	0.143
	12 weeks	0.14 ± 0.03	0.07 ± 0.01	
sE-selectin (ng/mL)	Baseline	50 ± 2	46 ± 2	0.001
	12 weeks	49 ± 2	53 ± 2	
VEGF (pg/mL)	Baseline	86 ± 14	126 ± 17	0.029
	12 weeks	152 ± 20	120 ± 17	
NO (μM)	Baseline	8.1 ± 0.6	9.5 ± 0.6	0.001
	12 weeks	10.6 ± 1.0	7.0 ± 0.3	

Values are mean ± SEM. TC, total cholesterol; HDL-C, high density lipoprotein cholesterol; LDL-C, low density lipoprotein cholesterol; hs-CRP, high-sensitivity C-reactive protein; sE-selectin, soluble E-selectin; VEGF, vascular endothelial growth factor; NO, nitric oxide.

**Figure 2:**
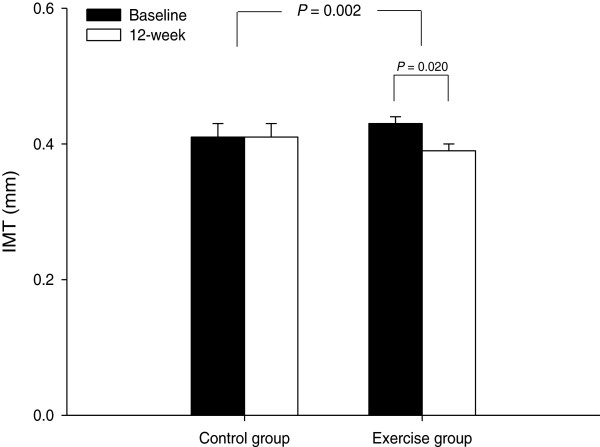
Mean (± SEM) carotid intima-media thickness (IMT) measured at baseline and 12 weeks in the control group (n = 14) and exercise group (n = 15).

For soluble E-selectin, vascular endothelial growth factor and nitric oxide concentrations, two-factor ANOVA revealed significant interactions (group × time), indicating that responses differed after 12 weeks between the control and exercise group (*p* = 0.001; *p* = 0.029; *p* = 0.001, respectively). For HDL-C and adiponectin, the group × time interactions were not significant. A significant group × time interaction was observed for carotid intima-media thickness (*p* = 0.002) (Figure 2). Carotid intima-media thickness decreased after 12 weeks of exercise in the exercise group (from 0.43 ± 0.01 to 0.39 ± 0.01 mm, *p* = 0.020).

**Figure 3:**
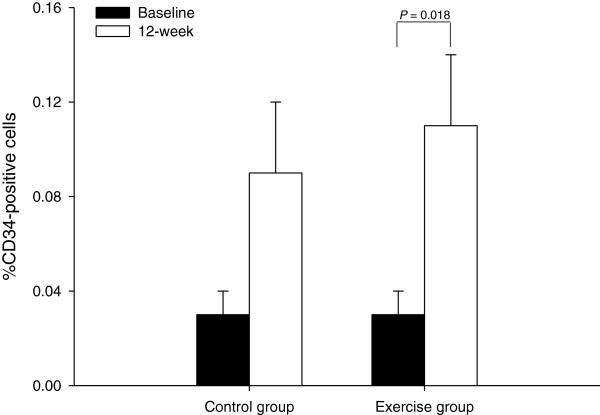
**Mean (± SEM) percentage of CD34**^**+**^**cells measured at baseline and 12 weeks in the control group (n = 14) and exercise group (n = 15).**

**Figure 4:**
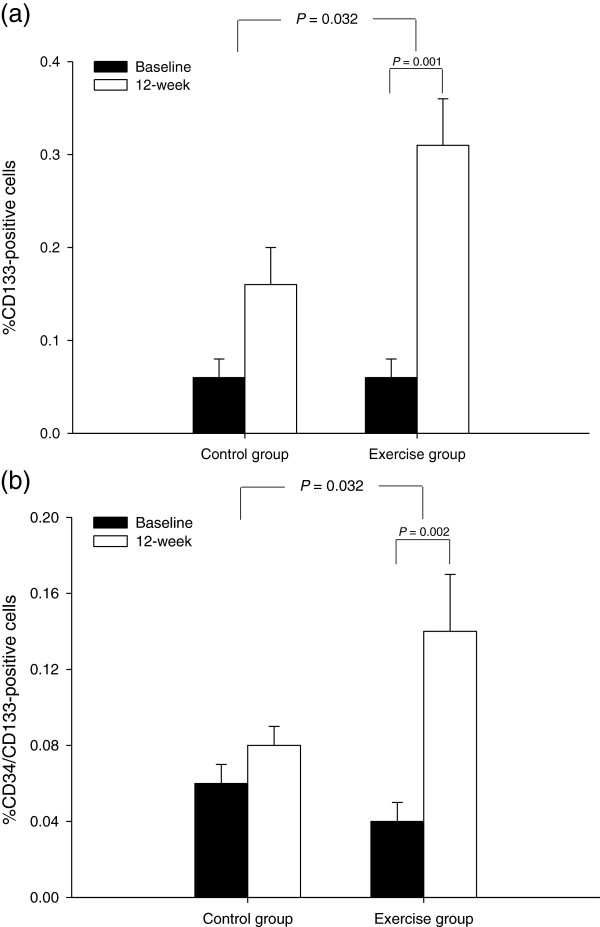
**Mean (± SEM) (a) percentage of CD133**^**+**^**cells and (b) percentage of CD34**^**+**^**/ CD133**^**+**^**cells measured at baseline and 12 weeks in the control group (n = 14) and exercise group (n = 15).**

There was no significant (group × time) interaction with percentage of CD34^+^ cells (*p* = 0.554) and no significant changes were observed in the control group (*p* = 0.063) (Figure 3) but there was significantly increased in percentage of CD34^+^ cells for the exercise group (from 0.03 ± 0.01 to 0.11 ± 0.03%, *p* = 0.018). Two-factor ANOVA revealed a significant interaction (group × time) effect for percentage of CD133^+^ cells (*p* = 0.032). Within-group analysis showed that the percentage of CD133^+^ cells was significantly increased in the exercise group after 12 weeks relative to baseline values (*p* = 0.001), but no significant changes were observed in the control group (*p* = 0.068) (Figure 4a). Two-factor ANOVA also revealed a significant interaction (group × time) effect for percentage of CD34^+^/CD133^+^ cells (*p* = 0.032). Within-group analysis showed that the percentage of CD34^+^/CD133^+^ cells was significantly increased in the exercise group after 12 weeks compared relative to baseline values (*p* = 0.002), but no significant changes were observed in the control group (*p* = 0.084) (Figure 4b). At baseline, there was no correlation between endothelial progenitor cells and carotid intima-media thickness (*r* = 0.365, *p* = 0.052). In addition, there was no correlation between changes in endothelial progenitor cells and carotid intima-media thickness (*r* = - 0.118, *p* = 0.329).

## Discussion

In this study, we investigated the effects of a 12-week after-school exercise programme on percentage of endothelial progenitor cells and other indices of cardiovascular health in overweight and obese children. Our programme, which consisted of a combination of aerobic and resistance training, resulted in an increase in the percentage of CD34^+^ and CD133^+^ cells in children who exercised. Similar findings have been recently reported by Zaldivar and colleagues [20], who showed that a brief bout of moderate to vigorous cycling exercise resulted in a significant increase in CD34^+^ (haematopoietic stem cells) in pubertal boys. However, to our knowledge these data provide the first evidence that combined aerobic and resistance exercise training may represent an effective strategy to improve vascular repair markers in overweight and obese children.

Endothelial progenitor cells promote angiogenesis and vascular repair, and enhance endothelial function [21]. Pluripotent haematopoietic stem cells also play a crucial role in vascular repair and angiogenesis [22], and several recent studies report the potential health benefits of haematopoietic stem cell mobilisation. Angiogenesis is a key factor in the training response to exercise, and mobilisation of haematopoietic stem cells may play a role in this process [23]. Our finding that percentage of CD34^+^ cells and percentage of CD133^+^ cells increased in overweight and obese children following participation in our exercise programme suggests that percentage of CD34^+^/CD133^+^ cells (endothelial progenitor cells) may mediate the tissue response to exercise. Although the change in the percentage of CD34^+^, CD133^+^ cells observed in control group were not significant, there was a trend towards an increased percentage of CD34^+^, CD133^+^ cells in the control group over the study period. This could be due to the mechanisms for endothelial progenitor cells related differences and it may be related to the different physiologic patterns of response to growth hormone and insulin-like growth factor-1 in pubertal children [24].

We found that our combined aerobic and resistance exercise programme caused an increase in endothelial progenitor cells in obese children. Aerobic exercise has been previously shown to increase the number of endothelial progenitor cells [9]; for example, Laufs and colleagues reported that 30 minutes of running at moderate or high intensity increased circulating levels of endothelial progenitor cells in healthy adults [10]. Similarly, Arnold and colleagues reported in a cross-sectional study that physical performance (peak oxygen uptake and maximum relative watt performance) in obese children is associated with endothelial progenitor cells [25]. In both of these previous studies, there was a close relationship between endothelial progenitor cells and physical activity. Based on our findings and those of previous studies, we speculate that this increase in the number of endothelial progenitor cells may be one component mediating the beneficial effect of exercise training on cardiovascular health in overweight and obese children. Resistance exercise is associated with increased circulating concentrations of vascular endothelial growth factor [26], which is an important mediator of endothelial progenitor cell activity. Thus, both aerobic and resistance exercise may positively contribute to endothelial progenitor cells and function. It is worth noting that exercise interventions that improve endothelial cell function are critical for defining effective approaches to prevent atherosclerotic cardiovascular disease associated with obesity, particularly in at-risk individuals. Our findings suggest that after-school exercise programmes such as the one we have tested may play an important role in preventing future disease in overweight and obese children.

Endothelial dysfunction is a risk factor for cardiovascular disease and an independent prognostic marker [27]. Therefore, maintaining normal endothelial function has become a major therapeutic goal. In this study, we showed that plasma vascular endothelial growth factor concentrations and nitric oxide concentrations were significantly increased and reduced plasma soluble E-selectin concentrations after a 12-week combined exercise programme. Vascular endothelial growth factor is an important mediator of angiogenesis and arteriogenesis, and production of nitric oxide is important for the release of endothelial progenitor cells from the bone marrow [28,29]. Furthermore, soluble E-selectin concentrations are influenced by nitric oxide availability [30]. It is possible that the observed increase in nitric oxide concentrations and endothelial cells is the result of an exercise-induced increase in nitric oxide bioavailability combined with stimulation of vascular endothelial growth factor. This may lead to increased nitric oxide generation by existing endothelial cells in response to the angiogenesis stimulated by the exercise training and to mobilisation of increased percentage of endothelial progenitor cells. However, in the present study, there were not significant correlations between endothelial progenitor cells and high-grade endothelial cell function markers (vascular endothelial growth factor, *r* = 0.005, *p* = 0.979; nitric oxide, *r* = 0.116, *p* = 0.389) (Data not shown).

Carotid intima-media thickness, a subclinical marker of atherosclerosis, is a strong marker of cardiovascular disease and is related to early atherosclerosis in children and adolescents [31,32]. It has recently been shown that aerobic exercise reduces carotid intima-media thickness in obese children [33,34] and that weight loss decreases carotid intima-media thickness [35]. Demonstrating the reversibility of early atherogenic changes we also found that carotid intima-media thickness decreased after 12 weeks of combined aerobic and resistance exercise in our study. In addition, Fadini and colleagues [6] have shown that lower numbers of circulating endothelial progenitor cells are associated with increased carotid intima-media thickness in middle aged adults. Thus, it is possible that exercise-induced mobilisation of endothelial progenitor cells is necessary to improve endothelial function. However, we failed to find the relationship between endothelial progenitor cells and carotid intima-media thickness. The possible reason for this inconsistency might be the age of the participants between studies. It may be also related to the different physiologic patterns of response for pubertal children [24].

In the present study, an after-school exercise programme led to beneficial changes in body composition and cardiorespiratory fitness in overweight and obese children. These findings are consistent with those of previous studies [36,37], which demonstrate that habitual activity is important to body composition and fitness. It is important to prevent obesity during development, and a regular exercise programme is considered an important preventative measure [38]. However, obese children who are home alone after school have limited time to be active outdoors and spend almost all of their time in sedentary activities, such as watching television, videos, or DVDs, playing video games, or using a computer [39,40]. Therefore, to increase their physical activity we conducted a regular after-school aerobic- and resistance exercise programme. Since children may be more open to changing in their lifestyles than adults, we anticipate that an early adjustment to regular exercise may bring about a persistent change in personal attitudes toward an active lifestyle. It is our hope that participation in our after-school exercise programme may result in increased habitual activity, at least in some children.

This study had some limitations. The time spent in activities outside of our study exercise programme was not measured, and could have potentially affected the results. In addition, differences in diet might also be a confounding factor [41]. Indeed, we have not controlled diet in both exercise and control groups throughout the study. Thus, although increased endothelial progenitor cells reported in the present study is likely due to the exercise per se, the influence of body fat reduction secondary to dietary changes may also potentially contribute. Finally, we have not examined the separate effects of the aerobic versus resistive exercise. It is not clear whether overweight children in our study would have the same response if participants perform one type of exercise.

## Conclusion

In conclusion, this study indicates that an after-school aerobic and resistance exercise programme is an effective lifestyle intervention strategy for improving endothelial progenitor cells in overweight and obese children. In addition, the continuation of the exercise programme for 12 weeks led to a decrease in body composition, an increase in maximal oxygen uptake and a reduction in carotid intima-media thickness. Therefore, these findings provide preliminary evidence that a combined training programme may represent an effective intervention strategy for improving vascular repair and endothelial cell function, leading to improved cardiovascular health in children.

## Competing interests

All authors declare that they have no conflicts of interest.

## Authors' contributions

JHP supervised data collection, data analysis, and wrote the first draft of the manuscript. MM provided guidance and assistance to JHP with data analysis, interpretation of data, edited the manuscript. YCK and HTP conceived the study and assisted with interpretation of data, edited the manuscript. EHK, JKP and KBP were involved in the recruitment of participants and assisted JHP with data collection. SRY and JWC performed the flow cytometry and assisted with the interpretation of data. YN and SKP conceived the study and provided guidance and assistance to JHP during this study. All authors read and approved the final manuscript. 

## Pre-publication history

The pre-publication history for this paper can be accessed here:

http://www.biomedcentral.com/1471-2431/12/111/prepub
